# Prevalence of Distant Metastases in High-Risk Operable Breast Cancer (HROBC) pT1-2 N2a or Higher at Diagnosis

**DOI:** 10.1007/s13193-024-02098-3

**Published:** 2024-10-07

**Authors:** Vani Parmar, Naveena Kumar AN, Nita S. Nair, Shalaka Joshi, Purvi Thakkar, Garvit Chitkara, Basila Ali, Varsha Gaikwad, Shabina Siddique, Vaibhav Vanmali, Palak Popat, Sneha Shah, Sangeeta Desai, Tanuja Shet, Meenakshi Thakur, Venkatesh Rangarajan, Rajendra Achyut Badwe

**Affiliations:** 1 Tata Memorial Centre and Homi Bhabha National Institute, Ernest Borges Road, Parel, Mumbai, 400012 India; 2 Breast Unit, Tata Memorial Hospital, Ernest Borges Road, Parel, Mumbai, 400012 India; 3 Breast Surgical Oncology, Punyashlok Ahilyadevi Holkar HN Cancer Institute of India, Mumbai, 400010 India

**Keywords:** Axillary nodal metastasis, Operable breast cancer, Metastatic workup

## Abstract

Current standard guidelines do not recommend a routine staging workup in early operable breast cancer (OBC) as the incidence of de novo metastasis is only 1–2%. Some of these patients are at high risk for relapse based on the higher axillary nodal burden. This prospective study evaluated the presence of de novo asymptomatic distant metastases in HROBC with pT1/2 N2a/N3 on upfront surgery. A single-centre study was carried out in upfront operated OBC patients with four or more axillary nodes positive after definitive surgery. Comprehensive metastatic workup was carried out, including an ultrasound abdomen, bone scan, and CT scan (thorax-abdomen-pelvis) or PET scan before initiating adjuvant treatment. If a distant disease was detected, the adjuvant treatment intent was tailored accordingly. The study accrued a prospective consecutive cohort of 97 women with pT1-2 N2a-3 during 2015–2018 operated upfront for OBC with cT1-2 N0-1. Forty percent of women were premenopausal, 54 (55.6%) had pN2a, and 43 (44.3%) had pN3 disease. Distant disease was seen in 8 of 97 women (8.24%) of these high-risk early cancers, 5 had oligometastatic denovo disease (5.15%), and 3 had polymetastatic (3.09%). Between the 2 groups, the pickup rate of distant disease was higher in pN3 (11.4%) as against pN2a (5.6%), *p* = NS. Only 3.09% of patients with extensive metastases were treated with palliative intent. The study shows potential to optimize the management of HROBC with heavy nodal disease identified post-primary surgery by selective staging investigations, adequate resource stratification, and thereby improved management, including modifying treatment plans early.

## Introduction

As per current guidelines, metastatic workup is not routinely recommended in women with clinically operable breast cancer (OBC) at presentation, as the incidence of asymptomatic metastasis is very rare in early stages, quoted to be 1–2% [1].

The 2019 Early Breast Cancer: ESMO Clinical Practice Guidelines [2] for diagnosis, treatment, and follow-up advise physical examination to be done as an assessment of metastases, and other tests are not routinely recommended unless initial high tumour burden, aggressive biology, or symptoms suggestive of metastases are present. Nodal disease burden is the most important prognostic factor, and patients with node-positive breast cancers are at a higher risk for developing relapses and distant metastatic disease. The detection rates of incident metastases increase with higher pN categories in early breast cancer as follows: 1.7% in pN1 (1–3 N +), 9.5% in pN2 (4–10 N +), and 13.5% in pN3 (> 10 N +) [3].

The National Comprehensive Cancer Network (NCCN) guidelines recommend a preoperative metastatic workup for stage I and II breast cancer patients only if symptomatic and routinely in large operable (LOBC) and locally advanced breast cancer (LABC) [4]. But no definitive recommendations are made for operable breast cancer patients who are found to have a heavy disease burden after upfront surgery, defined as N2/N3 disease or ≥ 4 positive axillary lymph node metastases on histopathology of axillary clearance. Currently, there is no compelling data to support a routine metastatic workup in these patients.

In recent times, there has been an increasing use and demand for PET-CT among women with breast cancer including those diagnosed at an early stage. The incidence is more common among women with node-positive disease or aggressive tumor biology, despite the lack of concrete evidence to support the same. In TNBC and Her2neu-positive biology, this is seen more often, especially if they are clinically node positive and chemotherapy is being planned before surgery, as poor responders in these subtypes have been shown to have a survival advantage with extended maintenance or change of systemic therapy [5, 6]. The PET-CT in these patients is not supported by any evidence or guidelines, although neo-adjuvant chemotherapy may be.

Upstaging on pathology demonstrates the fallacies of clinical staging. Approximately 15–30% of patients with clinical node-negative status (cN0) may get upstaged on pathology to N1 [7]. We often face the dilemma wherein upfront-operated breast cancer patients with an apparent good outcome may be found to have a high disease burden with higher nodal positivity on histopathology and, hence, a poorer outcome. The study done by Quiet et al. suggested that four metastatic nodes were an indicator of likely systemic disease [8]. Chu et al. reported stage 4 disease in 16% of OBC [9].

Investigating for asymptomatic metastases in pN2 and above inoperable breast cancer patients may prove to be resource-saving in case of metastatic disease being detected. The standard staging investigations included ultrasound abdomen-pelvis and bone scan, computed tomography (CT) thorax and abdomen, or PET CT scan [4].

## Aim

The current study is a single centre, non-randomized, prospective study aimed to detect the prevalence of de novo distant metastases in clinically early, operable breast cancer patients with pT1/T2 N2a or higher N stage early breast cancer, having a high nodal burden of 4 or more axillary nodes positive on final histopathology after their definitive surgery, with an aim to

- Identify the accurate clinical stage of disease in T1-2N2a and above by detecting subclinical de novo metastatic disease
- Evaluate a select subset of women with a clear cut-off value of high axillary node-positive early breast cancer, wherein a metastatic workup post hoc after surgery may be advised for appropriate resource management and staging.

The primary endpoint is the prevalence of stage IV disease detected on metastatic workup immediately after definitive surgery in pN2a node-positive early breast cancer, not otherwise meriting a metastatic workup.

## Inclusion Criteria

All patients meeting both of the following criteria, with

- Clinically T1-2 N0-1 operable breast cancer undergoing upfront surgery (no neo-adjuvant therapy)
- And on histopathology examination (HPE) have metastasis in 4 or more positive axillary lymph nodes

## Exclusion Criteria

- Locally advanced breast cancer (LABC)/ Large operable breast cancer (LOBC) warranting metastatic workup as a standard routine
- Previous interventions such as neo-adjuvant chemotherapy (NACT) or neo-adjuvant hormonal therapy (NAHT)

## Methods

The study was a prospective cohort study and accrued 97 eligible patients from November 2015 to July 2018. Study was carried out after institutional review board ethics approval with the due informed consent of the patient. Funding was provided from intramural funds at the institution.

During this time period, a total of 2800 operable breast cancer cases were registered and had upfront surgery as they were deemed to have operable breast cancer and did not receive neoadjuvant chemotherapy. Of these, 447 (16%) women fit the eligibility criteria on histopathology with pT1-2 pN2a or higher stage disease. None of them had required a metastatic workup before surgery as per the guidelines for early breast cancer and were all asymptomatic.

The current study screened consecutive 103 such patients who fulfilled the inclusion criteria; 97 eligible after informed consent were additionally evaluated by imaging for distant metastases by CT thorax and abdomen and bone scan or whole body PET scan. Patients without distant metastases on additional imaging were treated with standard adjuvant treatment protocol.

The patients, if detected with distant metastases (excluding oligometastasis), were managed as per standard stage 4 disease management guidelines and, hence, did not receive adjuvant radiation therapy. The option of treatment would, in these cases, now include chemotherapy (palliative) and radiotherapy only to distant site, as indicated. All oligometastatic cases were treated with curative intent.

### Statistical Methods

All the relevant patient and tumor characteristics as well as the results of the metastatic workup were noted. Comparison between 2 categorical variables was done with an unpaired *t*-test (chi-square/Fisher). Comparisons between 2 continuous variables were done with the Mann–Whitney or Wilcoxon test. A *p*-value of 0.05 was considered statistically significant. Results were analyzed with an aim to identify a cut-off of the number of positive lymph nodes beyond which patients are significantly upstaged to M1 disease.

## Results

Of the 103 screened women, 97 patients were accrued with pT1-2, N1-2a,3 disease and were included in the final analysis. All were consented prior to accrual.

The median age was 50 years, with pre/perimenopausal women making up 40.2% of the cohort, postmenopausal 42.3%, and post hysterectomy 17%. Of these, 64.9% underwent surgery in TMC, and the remaining 35.1% had undergone primary surgery elsewhere. A third of the cases had breast conservation surgery, and the remaining 70% had MRM (Table 1).

**Table 1:** Patient and tumor demographics

		**4–9 LNs positive (*n* = 54)**	** > = 10 LNs positive (*n* = 43)**	**Total**
Menopausal status	Pre + Peri	20	19	39
	Post	23	18	41
	Post hysterectomy	11	6	17
cT stage	T1	9	2	11
	T2	45 (83.3%)	41 (95.3%)	86
Surgery	BCS	17	13	30
	MRM	37 (68.5%)	30 (69.7%)	67
EIC	Positive	7	9	16
	Negative	41	28	69
	NK	5	6	11
LVE	Positive	37 (68.5%)	33 (73.7%)	70
	Negative	14	9	25
	NK	3	1	4
PNE	Positive	39	39	78
	Negative	11	2	13
ER	Positive	39	27	66
	Negative	15	16	31
PgR	Positive	30	23	53
				44
	Negative	24	20	
HER2	Positive	18 (33.3%)	10 (23.2%)	28
	Negative	29	24	53
	Equivocal	7	9	16

Majority (88.7%) had clinical T2 tumor, 11.3% had T1, and a median pT size of 3.15 cm. All were confirmed histopathologically as node-positive post-primary surgery, with 55.7% being pN2a (4–9 nodes positive), and the rest 44.3% had pN3 (with 10 or more positive axillary nodes) on the axillary clearance.

Biologically, these high nodal burden patients were HER2 expressing in 28.9%, with 16.5% HER2 equivocal but FISH not done; 18.5% were TNBC, 68% were ER positive, and 54.6% were PgR positive. Lymphovascular emboli overall were seen in 72.2% (slightly more in ≥ 10 LNs positive than in the 4–9 LNs positive group (73.7% vs 68.5%), and perinodal extension was detected in 85.7% of cases. Metastatic workup as per protocol was performed in all cases.

The metastatic staging detected the distant disease in 8 of these 97 patients; in other words, an 8.24% pickup of metastatic disease. The site of metastases and extent of distant disease were also assessed. There were 5 patients where the distant disease was detected in bone, and 3 others had liver as their distant site. Interestingly, 4 with bone disease and 1 with liver metastases (total 5/97, 5.15%) turned out to be low disease burden and qualified as oligometastatic disease. One other patient with bone and 2 patients with liver metastases (3/97, 3.09%) had multiple lesions and received palliative intent chemotherapy. As per protocol, the 5 patients with oligometastatic disease completed their adjuvant treatment, including radiation therapy. Conclusively, the detection rate of significant metastatic disease was only 3.09% in our study, referring to the 3 patients with multiple bone/liver metastases.

When correlated with regards to the number of nodes positive, 55.7% of patients had 4–9 nodes positive, and 44.3% had ≥ 10 LN positive. The relative detection rate of any distant metastases was 5.6% (3 of 53) in patients with 4–9 LN positive (1 bone and 2 liver) and in 11.4% (5 of 44) in patients with ≥ 10 LN positive (4 bone and 1 liver), but the difference was not statistically significant in the current study due to the small numbers (Table 2).

**Table 2:** Site of metastases by LNs positive

	**Site of metastasis**			
	**Liver**	**Bone**	**None**	Total
**4–9 LNs positive**	2	1	50	53
** > 10 LNs positive**	1	4	38	44
**Total**	3	5	88	97

Oligometastasis was detected in 5 cases (5.15%), and these were one patient in the 4–9 LN positive subgroup and 4 patients in the ≥ 10 LN positive. Polymetastatic disease (3.09%) was 2 in the 4–9 LN positive group and 1 in the ≥ 10 LN positive group (Table 3). The numbers are again too small to draw any definite conclusion. A definite cutoff could not, therefore, be identified beyond which there is a definite increase in the risk of distant metastatic disease. However, 10 or higher LNs positive does show a higher detection rate (11.4% vs 5.6%, *p* = NS), supporting a metastatic staging workup in this higher-risk subgroup before starting adjuvant therapy.

**Table 3:** Extent of metastases by LNs positive

	**Extent of metastasis**			
	**Oligo**	**Poly**	Nil	Total
**4–9 LNs positive**	1	2 (3.7%)	50	53
** > 10 LNs positive**	4	1 (2.2%)	38	44
**Total**	5 (5.15%)	3 (3.09%)	88	97

## Discussion

Standard NCCN guidelines so far do not recommend for routine metastatic workup in early breast cancer as the pickup rate is very low. Literature has suggested that only 1–2% of early operable breast cancer patients are detected with distant metastasis, and hence, it is not cost-effective for every patient to undergo evaluation of distant sites to detect metastases. Our intent was to identify a subset of patients within this early stage of disease with a higher risk of being detected with distant metastases, with already existing subclinical distant disease.

In the current study, in patients with the defined high-risk disease within early breast cancer, there was an 8.24% rate of detection of distant metastases which supports the role of metastatic workup in these select patients, helping to maintain resource stratification and utilization in early stage patients, correctly identifying such patients who would benefit most from adjuvant systemic and locoregional treatment. Of these, 5.15% were pre-existing oligometastatic disease that could be addressed upfront before starting adjuvant systemic therapy and therefore will not contribute to the apparent numbers that failed or progressed on systemic therapy if they were to be detected later in the course of treatment.

There was a multiple distant metastases pickup rate of 3.09% in patients who have 4 or more axillary LN positive. The numbers being small, the relatively higher risk subset of patients within the node positives could not be clearly identified. The ratio of the oligo to poly metastatic disease in the 4–9 LN + subgroup was 1:2, as compared to the subset with ≥ 10 LN + where it was 4:1; the difference was not statistically significant.

However, there is a clear trend towards doubling of risk of metastases (11.4% versus 5.6%, *p* = NS) when 10 or more LNs are positive in the axilla as against 4–9 LNs positive. This is similar to what is seen in literature, with the detection rates of metastases increasing with higher pN categories, pN1 (1–3 N +) with 1.7%, pN2 (4–9 N +) 9.5%, and pN3 (≥ 10 N +) with 13.5% distant metastases identification rate [3]. Although in our study we failed to have a clear cut-off value for the number of positive nodes to suggest metastatic workup as a standard of care, a detailed metastatic workup may be suggested in all cases that have 10 or more axillary nodal metastases in an otherwise early-stage breast cancer patient due to the rising trend seen. There are few other studies [8, 9] that have also evaluated natural history of breast cancer based on extent of axillary nodal burden.

One may argue that offering locoregional treatment in a patient with de novo metastatic disease may be detrimental as shown in various randomized clinical trials [10, 11], but evaluating for distant metastases in high nodal burden has not been recommended within any accepted guidelines for early breast cancer and thus is not standard of care.

Hence, a decision to decide post-hoc based on the axillary histopathology and nodal burden may not be unethical. On the other hand, it may actually help to identify correctly preexisting distant disease, prevent possible complications, in otherwise very high-risk disease, allow early distant intervention, and help improve the quality of life of such patients. In addition, this would help in better resource stratification and appropriate management of these cases.

Other point of debate is whether offering treatment to a distant site in an oligometastatic breast cancer improves outcome, if at all. The standard of care in oligometastatic disease has been so far to offer metastasis-directed treatment to distant sites as SBRT or surgical as indicated, with the aim to improve progression-free survival (PFS) and overall survival (OS), although there is now evolving evidence to the contrary in some small studies [12].

## Conclusion

The study provides valuable insight into the prevalence of asymptomatic distant disease in high-risk (≥ 4 LNs positive) breast cancer patients. Early breast cancer patients classically do not require a metastatic workup as the rate of detection of distant metastases is very low. However, a post-surgery workup may be advisable in selective higher-risk patients with pN3 disease with 10 or more LNs positive in the axillary nodes for identifying 11.4% of patients who may have distant site involvement. Although most of these may be oligometastatic, the same may also then be included in treatment. Early detection of metastatic disease could lead to improved treatment strategies and resource allocation, potentially enhancing patient outcomes in this higher-risk early breast cancer. The findings have the potential to optimize the management of high-risk early breast cancer as a separate entity, somewhere between the excellent results seen in early cancer and the locally advanced breast cancer with higher relapse rates and guarded prognosis.
